# The Influence of the Practice of the Daguerrotype Art

**Published:** 1853-08

**Authors:** 


*The influence of the practice of the Daguerreotype Art, upon the health of the Operator.*— Read by J. C. Morfit, M.D.

I wish to bring before the notice of the Society some points which I have not seen mentioned before. Being at a Daguerreotypist's about two weeks since, the operator was complaining of suffering from an abscess in the axilla which had troubled him for several days, and which he expected would, and which really did, burst that day. Upon enquiring as to the probable cause of its appearance, he said that he, and others engaged in the same business, were subject to swelling and enlargement of the glands all over the body, that many times the inguinal glands were enlarged and presented the appearance of venereal buboes, for which they would most likely be mistaken if the physician was not aware of the patient's calling, and its effects on the system. The appearance of these swellings is to be accounted for in the fact that the plates have to be coated with mercury, and as this is done in a small closet the operator is exposed fully to the fumes arising from the mercurial bath. The men, too, wet their hair to keep the dust and dandruff from flying on the plates, and hair by being thus moistened is more liable to retain the vapor. What effects the bromine may have I am unable to say, though I should think they were more confined to the respiratory organs. Seeing the bad effects of this calling and noticing the liabilities to cold in those engaged in it, the question naturally arises, what can be done to procure perfect immunity ? I leave this question open to the Society. But, with a little care, protecting the head by a cap, frequent ablutions and plenty of air, I should think the dangers would be much less.

